# Clinical pharmacogenetic analysis in 5,001 individuals with diagnostic Exome Sequencing data

**DOI:** 10.1038/s41525-022-00283-3

**Published:** 2022-02-18

**Authors:** Javier Lanillos, Marta Carcajona, Paolo Maietta, Sara Alvarez, Cristina Rodriguez-Antona

**Affiliations:** 1 Hereditary Endocrine Cancer Group, Human Cancer Genetics Programme, Spanish National Cancer Research Centre (CNIO), 28029 Madrid, Spain; 2 NIMGenetics, 28049 Madrid, Spain; 3 Centro de Investigación Biomédica en Red de Enfermedades Raras (CIBERER), Madrid, Spain

**Keywords:** Predictive markers, Pharmacogenomics, Next-generation sequencing

## Abstract

Exome sequencing is utilized in routine clinical genetic diagnosis. The technical robustness of repurposing large-scale next-generation sequencing data for pharmacogenetics has been demonstrated, supporting the implementation of preemptive pharmacogenetic strategies based on adding clinical pharmacogenetic interpretation to exomes. However, a comprehensive study analyzing all actionable pharmacogenetic alleles contained in international guidelines and applied to diagnostic exome data has not been performed. Here, we carried out a systematic analysis based on 5001 Spanish or Latin American individuals with diagnostic exome data, either Whole Exome Sequencing (80%), or the so-called Clinical Exome Sequencing (20%) (60 Mb and 17 Mb, respectively), to provide with global and gene-specific clinical pharmacogenetic utility data. 788 pharmacogenetic alleles, distributed through 19 genes included in Clinical Pharmacogenetics Implementation Consortium guidelines were analyzed. We established that Whole Exome and Clinical Exome Sequencing performed similarly, and 280 alleles in 11 genes (*CACNA1S*, *CYP2B6*, *CYP2C9*, *CYP4F2*, *DPYD*, *G6PD*, *NUDT15*, *RYR1*, *SLCO1B1*, *TPMT,* and *UGT1A1*) could be used to inform of pharmacogenetic phenotypes that change drug prescription. Each individual carried in average 2.2 alleles and overall 95% (*n* = 4646) of the cohort could be informed of at least one actionable pharmacogenetic phenotype. Differences in variant allele frequency were observed among the populations studied and the corresponding gnomAD population for 7.9% of the variants. In addition, in the 11 selected genes we uncovered 197 novel variants, among which 27 were loss-of-function. In conclusion, we provide with the landscape of actionable pharmacogenetic information contained in diagnostic exomes, that can be used preemptively in the clinics.

## Introduction

Adverse Reactions to Drugs (ADRs) and therapeutic failure are major public health care problems^1,2^. Over 200 genes have been associated with therapy response and are recognized as pharmacogenetic genes^3^ and between 80 and 99% of individuals are estimated to carry genetic variants that modify drug response^4,5^. Pharmacogenomic information is included in drug labels and several clinical guidelines have been elaborated to adjust drug prescription to the genetic background of the individual (e.g. by the Clinical Pharmacogenetics Implementation Consortium-CPIC or by the Dutch Pharmacogenetics Working Group-DPWG)^6–9^. However, the implementation of pharmacogenetics in the clinics is still low.

Genotyping-based technologies are useful strategies for reactive pharmacogenetics, by guiding the use of particular medications or explaining specific ADRs^10–13^. They also promote preemptive pharmacogenetics^4^, but the recent explosion of next-generation sequencing (NGS) techniques creates a unique opportunity to accelerate this field^14^. Whole exome sequencing (WES) or whole-genome sequencing (WGS) are suitable approximations for repurposing available sequencing data and report pharmacogenetic actionable alleles^4,10,14–17^, while they can also uncover novel potentially relevant variants^18,19^. The accuracy of NGS for detection of pharmacogenetic variants is elevated, showing high concordance with genotyping techniques^10,14,17^, and customized pharmacogenetic NGS panels and WGS can resolve the full set of actionable pharmacogenetic alleles^17,20,21^, including copy number variations (CNVs) in important pharmacogenes such as *CYP2D6*^14,18,21–26^. Exome sequencing is limited by design (i.e. lack of non-coding regions, low resolution for CNVs, and incomplete HLA-typing accuracy), but at the moment, the large-scale NGS techniques most commonly used for diagnosis are WES together with the so-called Clinical Exome Sequencing (CES), which are smaller designs targeting disease-causing genes (typically, 12–17 Mb compared to 50–60 Mb of WES)^27–29^. Diagnostic WES and CES provide an extraordinary opportunity to repurpose this data to recover actionable pharmacogenetic information and boost preemptive pharmacogenomic testing.

After some pioneer studies with small sample sizes and/or lacking phenotype assignments^14,15,17^, two recent large biobank studies used research-oriented data to compare the pharmacogenetic allele resolution of genotyping arrays, WES and WGS^10^, and to investigate the frequency of pharmacogenetic alleles in the WES of 50,000 individuals in the UK Biobank^30^. Regarding diagnostic WES, a study in >1500 individuals investigated the feasibility of extracting 42 selected variants in 11 pharmacogenes^16^, and two additional studies with >1000 individuals explored the secondary use of WES for pharmacogenomics in specific patient groups^31,32^. However, these studies have missed some clinically relevant pharmacogenes (e.g. *CACNA1S*^10,14–16,31^, *CYP2B6* and *UGT1A1*^31,32^, *CYP4F2* and *G6PD*^15,16^, *NUDT15* and *RYR1*^14–16^), they are population-specific^10,30,32^, and none has investigated the potential utility of CES. Therefore, a systematic analysis including all pharmacogenetic variants defined as actionable by international consensus, within a routine clinical diagnostic WES context, is still lacking.

In this study, we performed a comprehensive pharmacogenetic analysis in 5001 individuals that underwent WES or CES for genetic diagnosis in Spain and Latin American countries, by extracting and analyzing the 788 pharmacogenetic alleles defined in any of the 25 Clinical Pharmacogenetics Implementation Consortium (CPIC) guidelines currently available. In addition, we investigated differences in allele frequencies among populations and extracted novel loss-of-function (LOF) variants to estimate their contribution to the different pharmacogenetic phenotypes.

## Results

### Study cohort

We retrospectively collected pharmacogenetic data from 5001 unrelated individuals that underwent routine WES or CES aimed at medical diagnosis of diverse hereditary conditions (NIMGenetics, Madrid, Spain; between July 2017 and May 2019) using two frequently used commercial library kits (*n* = 4002 WES and *n* = 999 CES; see Methods section for platform details). 53.8% of the cases were females (*n* = 2690), 44.6% males (*n* = 2231), and for 1.6% (*n* = 80) gender was unknown. Regarding the country of origin, 2862 individuals (57.2%) were from Spain, 2016 (40.3%) were from Latin America (1342 Colombia, 568 Brazil, 92 Mexico, 11 Ecuador, 1 Argentina, 1 Chile and 1 from an unknown location in Latin America), 40 (0.01%) were from other diverse countries and 83 individuals (1.7%) were from an unknown location.

### Selected pharmacogenetic alleles with clinical actionability

**Fig. 1:**
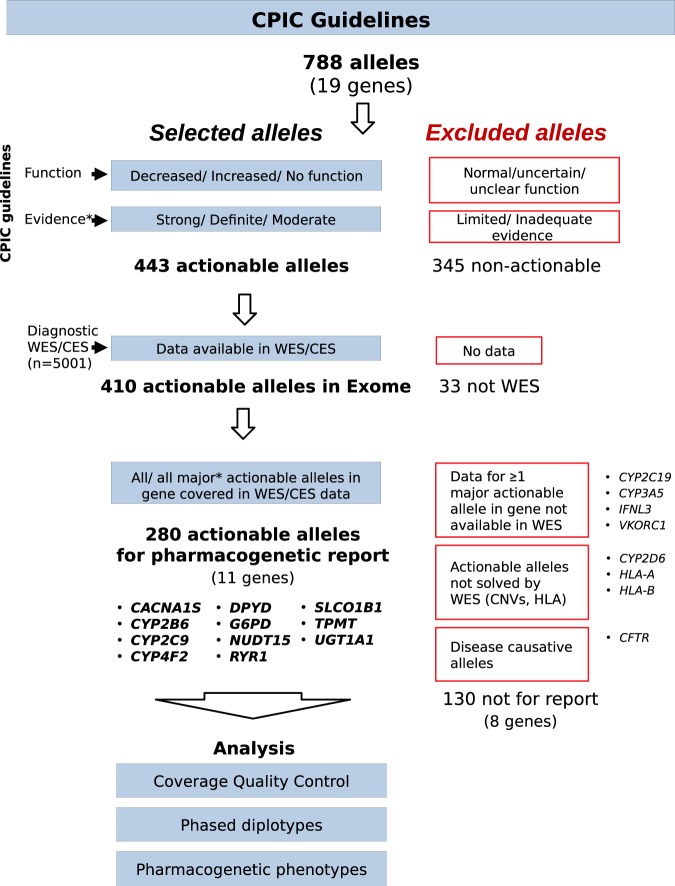
Workflow diagram describing how actionable alleles and pharmacogenes were selected based on CPIC clinical guidelines. *Evidence: “evidence level” is obtained from CPIC “allele functionality” tables. This information is only available for *CYP2C19*, *CYP2C9,* and *DPYD* genes.

Figure 1 summarizes the workflow that was followed for allele selection. All pharmacogenetic alleles described in any of the 25 available CPIC clinical guidelines (https://cpicpgx.org/guidelines/; accessed in July 2021) were selected for the study (788 alleles distributed in 19 genes). We filtered out those with no application in the clinics (with normal function, with unclear/uncertain/unknown impact in function or with a limited/inadequate strength of evidence, when available; 345 alleles discarded and 443 retained), then we identified alleles defined by intronic, intergenic or upstream regions variants not present in the exome data (5001 exomes analyzed; 33 alleles discarded and 410 retained). At this filtering step, no differences were observed between WES and CES. At this point, we reasoned that 8 genes (*CYP2C19*, *CYP3A5*, *IFNL3*, *VKORC1*, *CYP2D6*, *HLA-A*, *HLA-B,* and *CFTR*) could not be fully informed because (i) a major actionable allele was absent in exome data (CYP2C19*17-promoter region, CYP3A5*3-deep intronic, *IFNL3* rs12979860-non-coding region and *VKORC1* rs9923231-promoter region) and this would lead to an incomplete pharmacogenetic report with no associated clinical recommendation; (ii) an important actionable allele is a Copy Number Variant (*CYP2D6*) or is a HLA allele (*HLA-A* and *HLA-B*) which cannot be accurately resolved by exome data. Partial gene deletions in *CYP2B6* (CYP2B6*29 and CYP2B6*30 alleles) cannot be detected by exome, but due to their rarity in tested populations (<1% in African American and Asians individuals)^33,34^, this gene was not excluded from the analysis^13^; and (iii) these were disease causative alleles (*CFTR*) that are out of the objectives of preemptive pharmacogenetics. Therefore, we filtered out all the alleles in these 8 genes, and retained a total of 280 alleles distributed in 11 pharmacogenes (*CACNA1S*, *CYP2B6*, *CYP2C9*, *CYP4F2*, *DPYD*, *G6PD*, *NUDT15*, *RYR1*, *SLCO1B1*, *TPMT,* and *UGT1A1*) that were considered suitable to generate a comprehensive exome-based pharmacogenetic report and were kept for the analysis (Fig. 1).

### Sequencing depth of coverage data and quality control

We performed a sequencing coverage quality control (QC) of all genetic loci required to resolve the 280 actionable alleles (Supplementary Fig. 1A). All interrogated loci were covered by both exome designs, despite we observed some differences between WES and CES for some genes. In CES there was a lower coverage in *CACNA1S* caused by the variant rs772226819 (40% of samples covered <40x, although 99.5% had >20x). In WES there was a lower coverage in *TPMT* at variant rs1142345, defining TPMT*3C and TMPT*3A alleles (80% of samples <40x, although 90% had >20x). The gene with the lowest coverage was *UGT1A1*, especially in CES, due to the indel variant rs34983651, which defines the alleles UGT1A1*28, *36, and *37 (the percentage of samples covered >20x were 93 and 34% for WES and CES, respectively). Of note, *CYP2B6* and *CYP2C9*, with the lowest level of sequence uniqueness (Supplementary Fig. 1B), did not show lower coverage nor evidence of potential calling errors in our analysis. Overall, an average coverage >20x was observed for all loci (except for rs34983651) in 99.3% of the samples, indicating that repurposing of diagnostic WES and CES data for clinical pharmacogenomics is a suitable approximation for the selected genes.

### Pharmacogenetic diplotypes

**Fig. 2:**
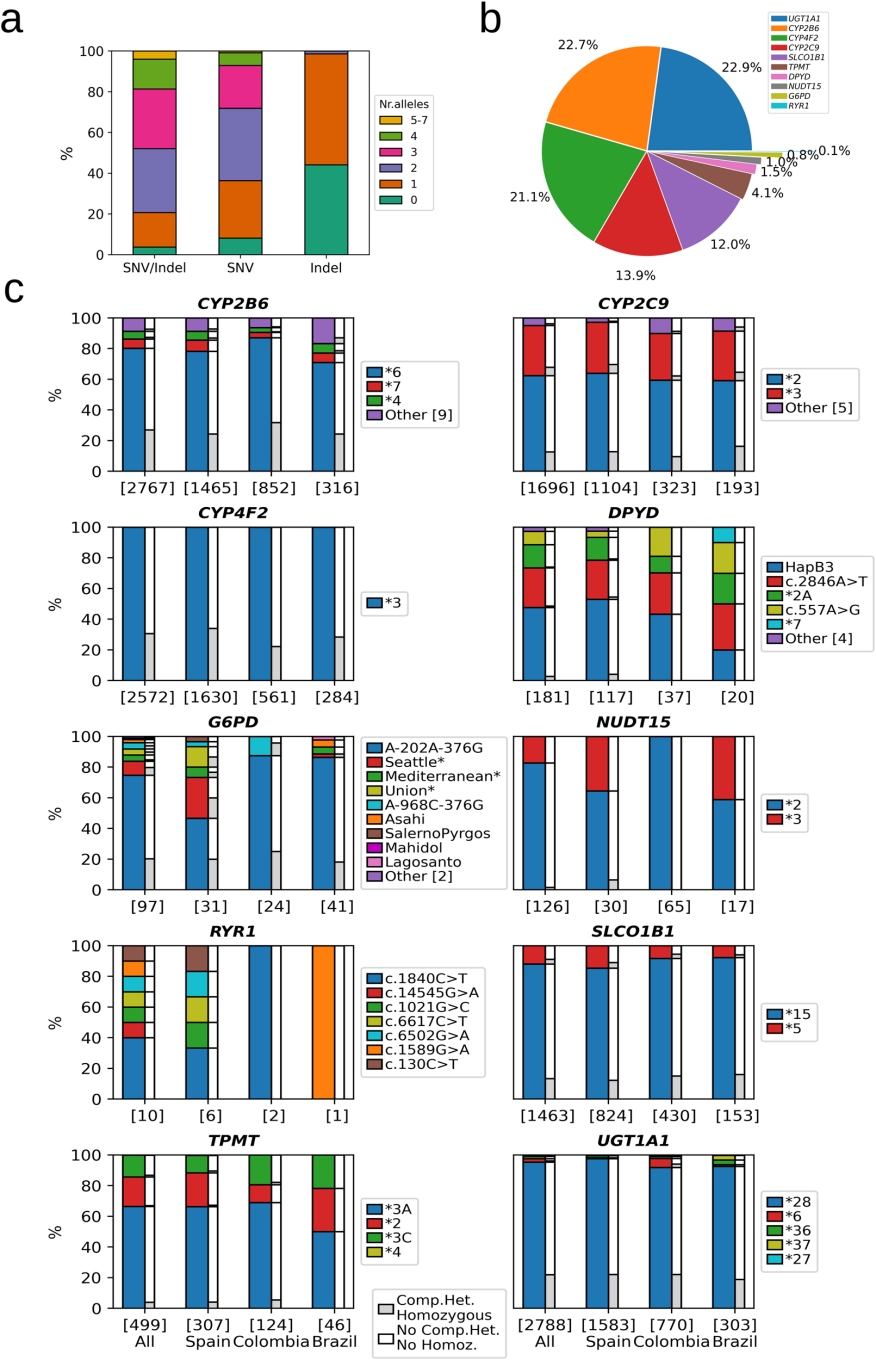
Alleles distribution and population differences. **a** Colored stacked bar plots show the percentage of individuals which carry none, one, or multiple alleles for SNVs and indel variants. **b** Pie chart showing the percentage of alleles found per gene out of the total number of actionable alleles (*n* = 12,309) in the whole cohort (*n* = 5001 individuals). **c** For each actionable gene, a group of four stacked bar plots depicts alleles distributions within all (*n* = 5001) individuals, the Spanish, Colombian, and Brazilian subgroups, respectively. Y-axis labels represent the number of carriers in each specific group. For each allele depicted, the fraction of compound heterozygous (CH) and homozygous (Homoz) individuals is represented in gray bars versus remaining individuals in white bars. “Other” indicates low prevalent alleles represented together. The full list of alleles per gene can be found in Supplementary Data 1. Some allele names in *G6PD* gene (marked with an asterisk) have been shortened: Seattle Lodi Modena FerraraII Athens-like: Seattle*, Mediterranean Dallas Panama‚Sassari Cagliari Birmingham: Mediterranean*, Union Maewo Chinese-2 Kalo: Union*, G6PDA-968C-376G: A-968C-376G.

After variant genotype to allele diplotype conversion, 96.4% (*n* = 4823) of individuals harbored one or more actionable alleles (Fig. 2a). The average number of actionable alleles per individual was 2.2, with 6 individuals having 7 actionable alleles. Most individuals (79.3%) had 2 or more pharmacogenetic alleles, which illustrates the high pharmacogenetic heterogeneity (Supplementary Fig. 2), and 55% of the cohort carried either one (*n* = 2735) or two (*n* = 11) alleles defined by indel variants (Fig. 2a). Regarding the different genes involved, *UGT1A1* and *CYP2B6* were the two genes that contributed the most to the total alleles called, followed by *CYP4F2*, *CYP2C9,* and *SLCO1B1*, while a low number of alleles were observed for *TPMT*, *DPYD*, *NUDT15*, *G6PD,* and *RYR1*, and no individuals were found to carry any *CACNA1S* actionable allele (Fig. 2b).

The most frequent alleles in each gene are shown in Fig. 2c, for the whole cohort and for the largest populations analyzed (Spain, Colombia, and Brazil), together with the frequency in which those alleles are found in homozygosity or compound heterozygosity (gray bars in Fig. 2c). In *CYP4F2* and *NUDT15* only one or two actionable alleles were detected (CYP4F2*3, NUDT15*2, and NUDT15*3, respectively). In *SLCO1B1*, *UGT1A1*, and *CYP2B6*, one allele was far more frequent than the rest (SLCO1B1*15, UGT1A1*28, and CYP2B6*6, respectively), with *CYP2B6* having a significant proportion of less frequent alleles. In *TPMT*, the most common allele was TPMT*3A, followed by TPMT*2 in Spain or TPMT*3C in Colombia. In *CYP2C9*, the dominating alleles were CYP2C9*2 and CYP2C9*3, but additional minor alleles were detected, especially in Latin American population (10.6% and 9.9% in Colombia and Brazil versus 4.2% in Spain). In *DPYD*, the two most frequent alleles were *DPYD* HapB3 and *DPYD* c.2846A > T across the populations studied, followed by DPYD*2A in Spain and by c.557A > G in Colombia and Brazil. *RYR1* and *G6PD* genes were the more heterogenous genes, with diverse and low prevalent alleles across populations, in the case of *G6PD* with -202A-376G being the most common allele. The full list of alleles per individual is provided in Supplementary Data 2.

We compared the frequency of variants found in Spain and Latin America and the corresponding gnomAD population (Supplementary Fig. 3). Among high-frequency variants with differences, *SLCO1B1* rs2306283 (present in several *SLCO1B1* alleles) had the highest frequency in Brazil, *CYP4F2* rs2108622 (*3) in Spain and *CYP2B6* rs3745274 and rs2279343 (*6) in Colombians. Among medium and low-frequency variants, several *G6PD* variants had a large variability among populations, *TPMT* rs1800462 (*2) had the highest frequency in Spain and *DPYD* rs115232898 (c.557A > G) had higher frequencies in our populations (i.e. 0.00087 versus 0.000053 for Spain and gnomAD NFE; 0.003 versus 0.00078 for Latin American countries and gnomAD AMR) suggesting a contribution from Sub-Saharan Africa in our cohorts^35^. Differences with CPIC European and Latino allele frequency data is presented in Supplementary Fig. 4.

### Pharmacogenetic phenotype analysis

Allele diplotype to phenotype conversion revealed that 4646 individuals (95.1%) had a clinical actionable pharmacogenetic phenotype that could be reported based on exome data (Supplementary Fig. 5B). The proportion of individuals with an actionable phenotype differed according to the gene, with the highest numbers corresponding to *UGT1A1*, *CYP2B6*, and *CYP4F2*, followed by *CYP2C9* and *SLCO1B1* (Table 1 and Supplementary Fig. 5B). When only the more extreme phenotypes were considered (those in which an alternative drug is recommended due to an increased risk of severe/fatal toxicity, i.e. *CYP2C9* PM (Poor Metabolizer), *DPYD* PM, *G6PD* deficient, *NUDT15* PM, *RYR1* MHS (Malignant Hyperthermia Susceptibility) carriers, *SLCO1B1* LF (Low Function), and *TPMT* PM), these were present in 5.5% of the individuals.

**Table 1:** Pharmacogenetic phenotypes in the population.

Gene	Phenotype^a^	Activity score	Total(*n* = 5001)	Spain(*n* = 2862)	Latin America(*n* = 2016)	Colombia(*n* = 1342)	Brazil(*n* = 568)
			Percentage of the population				
*CACNA1S*	Non-MHS	–	100	100	100	100	100
	MHS		0	0	0	0	0
*CYP2B6*	NM	–	44.7	48.8	38.75	36.5	44.4
	IM		42.1	39.6	45.7	47.8	41.5
	PM		8.9	7.2	11.1	12.1	8.6
	RM		4.3	4.3	4.4	3.6	5.3
	URM		0.08	0.10	0.05	0	0.18
*CYP2C9*	NM	2	77.7	74.5	82.8	84.1	79.0
	IM	1.5	20.2	23.1	15.8	14.9	18.8
		1	0.28	0.24	0.4	0.30	0.18
	PM	0.5	1.5	1.7	0.9	0.6	1.8
		0	0.3	0.4	0.1	0.07	0.18
*CYP4F2*^b^	NM	–	46.9	41.3	54.8	56.6	49.5
	IM		43.1	46.1	38.9	37.7	42.2
	PM		10.0	12.6	6.3	5.7	8.3
*DPYD*	NM	2	96.4	95.9	96.9	97.2	96.5
	IM	1.5	2.9	3.2	2.5	2.5	2.5
		1	0.70	0.80	0.6	0.30	1.06
	PM	0.5	0.02	0.04	0	0	0
		0	0	0	0	0	0
*G6PD*	Normal	–	99.4	99.5	99.3	99.4	98.8
	Deficient		0.6	0.5	0.7	0.6	1.2
	Deficient (CNSHA)		0	0	0	0	0
*NUDT15*	NM	–	97.5	99.0	95.5	95.2	97.0
	IM		2.5	1.0	4.5	4.8	3.0
	PM		0.02	0.03	0	0	0
*RYR1*	Non-MHS	–	99.8	99.8	99.8	99.9	99.8
	MHS		0.20	0.21	0.20	0.15	0.1
*SLCO1B1*	NF	–	70.6	71.0	69.6	68.0	72.7
	IF		26.8	26.5	27.5	28.9	24.6
	LF		2.6	2.5	2.9	3.1	2.6
*TPMT*	NM	–	90.0	89.3	90.0	90.8	91.7
	IM		9.7	10.4	8.8	8.9	8.3
	PM		0.26	0.31	0.2	0.30	0
*UGT1A1*	EM	–	45.2	45.6	1.1	43.4	48.4
	IM		48.1	47.6	48.8	50.1	46.1
	PM		6.7	6.8	6.2	6.5	5.5

*MHS* malignant hyperthermia syndrome, *NM* normal metabolizer, *IM* intermediate metabolizer, *PM* poor metabolizer, *CNSHA* congenital non-spherocytic hemolytic anemia, *NF* normal function, *IF* intermediate function, *LF* low function, *EM* extensive metabolizer.

^a^Phenotypes according to CPIC guidelines: MHS, NM, IM, PM, CNSHA, NF, IF, LF, and EM.

^b^CYP4F2 phenotypes definitions (NM, IM, and PM) are not provided by CPIC guidelines.

Comparing the phenotypes of Spanish and the Latin American individuals, some statistically significant differences were observed in four genes (Table 1 and Supplementary Fig. 5C). For example, *NUDT15* Intermediate Metabolizer (IM) phenotype was higher in Latin America than in Spain (4.5% versus 1%, *p* < 0.00001; Supplementary Fig. 5C; and Table 1), while CPIC indicates frequencies of 8% and 0.8% for Latino and in European populations, respectively^36^. The combination of *TPMT* and *NUDT15* phenotypes resulted in 15 individuals being IMs for both genes, the majority being from Latin America (0.5% versus 0.1% in Spain).

### Novel Loss-of-Function variants

**Fig. 3:**
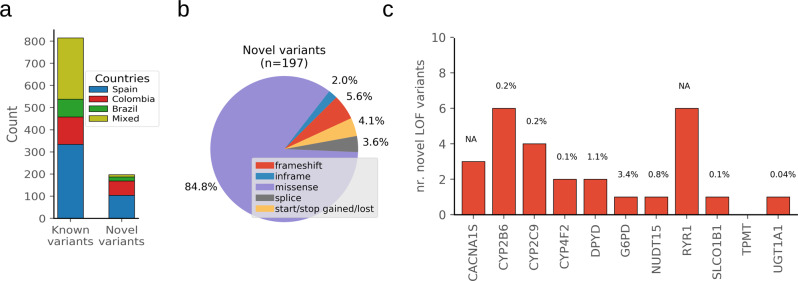
Discovery of novel variants. **a** Two stacked bar plots showing the number of known variants (left, variants reported in gnomAD or dbSNP databases) and novel variants (right, not reported in these databases). Blue, red and green stacked bars represent variants found exclusively in Spanish, Colombian, or Brazilian individuals, respectively, and yellow for variants found in a mixture of individuals from different countries. **b** Pie chart summarizing the fraction of different novel variants (missense, in-frame, frameshift, splice, or start/stop gained/lost). **c** Bar plots representing the number of novel LOF variants per gene. The percentages over each bar is an estimation of the contribution of the novel LOF in each gene over the total number of actionable alleles previously found. LOF variants in *CACNA1S* and *RYR1* have not been associated with increased risk of malignant hyperthermia, thus, the contribution of them to this phenotype is not applicable (NA).

In the eleven pharmacogenes analyzed in the exomes, we retrieved a total of 1012 variants, 19.5% of which were novel (167 missense, 11 frameshift, 7 canonical splice site, 8 stop gain, and 4 inframe variants) (Fig. 3a, b and Supplementary Data 3). As expected, the average allele frequency of the novel variants was lower than that of the known variants (0.00025 and 0.028, respectively, *p* value = 0.02) and 99% of the novel variants were found in one single country (either Spain, Colombia or Brazil), compared to 71% for known variants. Differently from the novel missense variants, which have an unknown effect in protein activity, LOF variants are expected to lead to a non-functional protein. The 26 novel LOF variants found were estimated to increase in average 0.54% the total number of actionable alleles, with a greatest impact on *G6PD* and *DPYD* and the lowest in *UGT1A1* and *TPMT* (3.4%, 1.1% and 0.04%, 0%, respectively; Fig. 3c). It is important to note that for the calcium release channels *RYR1* and *CACNA1S* the nonsense variants found have unknown significance, as only specific missense variants contribute to MHS. The full list of known and novel variants found in the selected list of pharmacogenes can be found in Supplementary Data 3.

## Discussion

Repurposing diagnostic exome data for clinical pharmacogenomics has the potential to globally change drug prescription. WGS is taking the lead for genomics medicine^37^, but WES and CES provide with an invaluable resource^38–40^ and a unique opportunity to implement pharmacogenetics, since they represent a competitive option among the large-scale NGS techniques used in the clinic^41,42^. In this study, by performing a systematic analysis of all pharmacogenetic alleles included in CPIC guidelines in 5001 exomes, we illustrate the pharmacogenetic actionable landscape contained in routine diagnostic WES and CES data.

Analyzing the 19 pharmacogenes included in CPIC guidelines revealed that 11 of these genes can be comprehensively informed (*CACNA1S*, *CYP2B6*, *CYP2C9*, *CYP4F2*, *DPYD*, *G6PD*, *NUDT15*, *RYR1*, *SLCO1B1*, *TPMT,* and *UGT1A1*) using WES or CES data. These genes are included in eight guidelines that describe relevant changes in therapeutic management of patients treated with volatile anesthetic agents and succinylcholine, antiretroviral therapy with efavirenz and atazavir, nonsteroidal anti-inflammatory drugs, fluoropyrimidines and thiopurines, rasburicase, and statins (see Supplementary Data 4). In addition, phenytoin and warfarin guidelines are partially covered: in the first case, informing about the recommended drug doses but missing the identification of patients with increased risk of hypersensitivity reactions^43,44^ and in the second case, missing *VKORC1* genotype information precludes comprehensive estimation of warfarin dose^44^. Thus, the pharmacogenetic information that is easily retrieved from exome data, is crucial to prevent drug adverse reactions (e.g. life-threatening malignant hyperthermia when treated with inhalation anesthetics or severe/fatal drug toxicity when treated with fluoropyrimidine drugs) and to guide drug dosing adjustments (e.g. to stabilize phenytoin plasma concentration within the targeted therapeutic range). Although data for seven relevant pharmacogenes (*CYP2C19*, *CYP3A5*, *CYP2D6*, *HLA-A*, *HLA-B*, *IFNL3*, *VKORC1*) could not be retrieved from exome data, the implementation of pharmacogenetics in the clinics is still low and repurposing routinely generated diagnostic WES/CES data to provide, without laboratory testing, the actionable phenotypes of 11 crucial pharmacogenes, is a step forward in preemptive pharmacogenetics.

Repurposing exome data for clinical preemptive pharmacogenomics requires the same QC that applies to any other genetic diagnostic test. To ensure this, first, we performed a depth of coverage analysis for each pharmacogenetic actionable allele and retrieved the genotype information in all loci. This step avoids misleading genotype data derived from low coverage. In the 11 selected genes, 99.3% actionable variants were covered at a standard diagnostic coverage threshold of 20x in both WES and CES data^45^. The genetic loci of the variant rs34983651, which informs UGT1A1*28, *36, and *37 alleles, was the only exception as it achieved a lower coverage in the CES data, that would result in *UGT1A1* gene exclusion in a substantial number of cases (22%). Some previous studies marked UGT1A1*28^16^ as a low-coverage allele also in WES, but we and others^21^ have reported them with high quality. Differences are probably due to technical reasons related to the panel design. Furthermore, the performance of other exome platforms for this position located outside the coding region might differ, marking this indel as a complex variant that requires specific analysis. Second, indel variants are an additional bioinformatic challenge and they constitute 21 (7%) of the actionable pharmacogenetic alleles interrogated. Thus, we manually reviewed them from the variant calling output, recovering high frequency (e.g. UGT1A1*28, *37, and *36) and low-frequency indels (e.g. CYP2C9*6 and DPYD*7). This step was necessary to avoid missing indel variants that were called differently due to reasons such as read sequence alignment^30^ (e.g. UGT1A1*28 can be called as C > CAT or CAT > CATAT). Indel normalization or a robust characterization of indel annotation by the variant callers could be strategies to automate this process. Third, diplotype translation according to CPIC definitions required manual curation for ambiguous calls and haplotype phasing, notably in *CYP2B6*, *TPMT*, and *G6PD*. These three highlighted issues are being addressed by novel bioinformatic approaches^30,46^ to facilitate automatization. In addition, although WGS and long-read sequencing face their own technical problems (e.g. coverage issues), repurposing their data for preemptive pharmacogenomics may help to overcome some issues derived from exome sequencing^7,19,46–50^.

Ancestry data is relevant for pharmacogenetic implementation, as several actionable alleles are subjected to important ethnic differences^51^. International efforts are contributing to expand country-based pharmacogenetics data^10,16,30,32^. However, in comparison to countries with national genomics medicine initiatives, the access of Latin American countries to NGS is still limited^11^. In this study, we compared variant frequencies in Spain, Colombia and Brazil and evaluated deviations from their closest gnomAD population, as this database is frequently used as reference in genetic studies^35^. Relevant differences included the variants defining CYP2B6*6, CYP4F2*3 and *DPYD* c.557 A > G alleles; which had higher frequencies in our studied populations than in reference populations previously reported^35,52,53^. Although our study is limited by using the country for the diagnostic test rather than ethnic group data in the analysis, from a healthcare point of view it is the pharmacogenetic landscape in each country, with current population admixture context, what will be most relevant to design genetic testing.

We and others have previously estimated the contribution of NGS to the detection of novel variants in pharmacogenes^24,25,30^. In this study, we complement these studies analyzing diagnostic exome data, since novel LOF variants detected by NGS will change the pharmacogenetic phenotype classification of the individual. For the 11 pharmacogenes that can be informed using exome data, the novel LOF variants contributed in average 0.54% to the total of known actionable alleles. However, most of the novel variants detected were missense variants (87%; Fig. 3b), part of which will affect protein activity and will contribute to the variability in drug response. In silico protein activity predictions are improving^19^, however, these evidences are still insufficient for clinical application and, thus, these rare missense variants have to be classified as of unknown significance. Beyond their importance for pharmacogenetics implementation, the incremental use of Electronics Health Records combined with prospective and retrospective genomics data and the development of novel computations tools predicting variant consequences, will help to elucidate the clinical impact of these variants and will reinforce the use of NGS^13,40^.

In conclusion, the high number of actionable pharmacogene alleles carried by the individuals and the urgent need for safer and more efficient personalized treatments, support the implementation of pharmacogenetics in genomic medicine^12^. The exponential growth in large-scale NGS diagnostics, with exomes being the most widely used platforms, argue for repurposing these data for clinical pharmacogenetics. The landscape of high-quality pharmacogenetic information that can be extracted from exomes and used to adjust drug treatments according to international guidelines, together with population-specific allele variations and estimations of the contribution of novel variants identified by NGS, all provided in this study, will aid in removing barriers and facilitating the clinical implementation of pharmacogenetics.

## Methods

### Study cohort and Whole Exome Sequencing/Clinical Exome Sequencing

We retrospectively collected NGS data from 5001 unrelated individuals who had undergone genetic testing aimed at medical diagnosis of diverse hereditary conditions at NIMGenetics´s genetic laboratory (28108 Madrid, Spain; https://www.nimgenetics.com/). While per-sample NGS data and sensitive information had been previously anonymized, cohort information included gender, country of origin for NGS testing, and trio availability. DNA samples were not available. The study was approved by the ethics committee of the ISCIII (IRB nr. CEI PI 105_2020-v2). As the study was performed on a retrospective set of anonymized samples, the approval from the ethics committee was obtained without the requirement of a specific consent inform from each patient for this study.

DNA libraries were prepared using a standard-size WES kit “SureSelect Human All Exon v6 kit” 60 Mb (Agilent, Santa Clara, CA, USA) for 4002 individuals, or with a CES kit “SureSelect Custom Constitutional Panel 17 Mb kit” (Agilent, Santa Clara, CA, USA) for 999 individuals. Paired-end sequencing was performed in a NovaSeq 6000 S2 Flow Cell (Illumina, San Diego, CA, USA). Bioinformatics analysis for germline SNVs and indel variant calling was performed following GATK Best Practices (Haplotypecaller^54^) using the GRCh37 human reference genome. Minimum mapping quality threshold was set to 20 (*–minimum-mapping-quality 20*). Variants were annotated with an in-house pipeline to include information regarding variant-specific predicted effect (impact, conservation), population-specific variant frequencies (e.g. gnomAD), and clinical annotations from medical databases (ClinVar, HGMD, disease-specific).

### Selection of pharmacogenes and alleles

First, we selected all pharmacogenes included in CPIC guidelines (https://cpicpgx.org/guidelines/; July 2021; *n* = 788 alleles) and compiled only those alleles annotated with no function, decreased function, and increased function in the “Allele Clinical Functional Status” column within the “Allele Functionality table”. Alleles annotated as normal function, uncertain function, unclear function, not reported or unknown function, were not considered further. Of note, *CACNA1S* alleles (c.520 C > T and c.3257 G > A) were considered as actionable despite having “uncertain” allele functional status due to their link to MHS; *G6PD* functional alleles were defined by I-III /Deficient status; despite the absence of a *CYP4F2* CPIC Allele Functionality Table in CPIC guideline for warfarin, based on this guideline, CYP4F2*3 allele was considered for the analysis, while CYP4F2*2 was filtered out^43^. Second, we filtered out alleles supported by a limited/inadequate level of evidence (this information was only available for *CYP2C9*, *CYP2C19,* and *DPYD* at the “Strength of Evidence” column located in their “Allele Functionality tables”). Third, we identified the alleles that could not be resolved using Exome data (e.g. those defined by intronic or promoter variants, CNVs or HLA alleles). If a high impact allele, critical for pharmacogenetic phenotype group definition, was not available in Exome data (e.g. CYP3A5*3, CYP2C19*17, *VKORC1*-rs9923231, CYP2D6*5), the gene was deemed as not interpretative by exome sequencing and all alleles of this gene discarded for the analysis. Fourth, *CFTR* was removed from the analysis because the alleles included in its guideline are disease-causing and thus not within the objectives of preemptive pharmacogenetics. Supplementary Data 1 contains the full list of pharmacogenetic alleles and variants in the 11 genes included in the analysis, as well as a link and accession dates to CPIC “allele/ functionality” tables.

### Quality control analysis

All actionable pharmacogenetics alleles defined by SNVs were subjected to QC analysis ensuring a correct homozygous-reference genotype calling using GATK v4 Haplotypecaller^54^ in “-ERC BP_RESOLUTION” mode. This strategy forces genotyping all relevant genomic loci, regardless of the nucleotide in the reference sequence (reference or alternative variant). Coverage values were extracted from the “depth” (DP) field to assess the fraction of samples that failed to inform at these sites. Alleles containing indel variation were manually curated to prevent genomic coordinate mismatches between called indels and their description. We reviewed all insertions and deletions flanking the described genomic coordinates of clinically actionable indels by searching within reported coordinates plus padded (upstream and downstream) genomic intervals with the length of the actionable indel. Also, QC analysis at the positions for the indels was performed. We extracted mappability scores (24, 36, 40, 50, 75, and 100-mer window sizes) from UCSC *Alignability* Tracks (http://genome.ucsc.edu/cgi-bin/hgFileUi?db=hg19&g=wgEncodeMapability) for all genomic loci included in this study.

### Haplotype and phenotype assignment

We obtained the pharmacogenetic information of the 280 CPIC actionable alleles (Supplementary Data 1) by leveraging genotype information obtained after the QC method described in the previous section. We calculated all possible actionable alleles according to the genetic variation and following the “Allele Definition Tables” provided in the CPIC guidelines. Similarly to the procedure described by McInnes et al^30^, if the defining genetic variation for one star allele was a proper subset of those for another star allele, the matching star allele with the greatest number of variants was reported. This situation was necessary for some alleles in *SLCO1B1* (*5 and *15), *CYP2B6* (*4, *6, *7, *8, *9, *13, *18, *20, *22, *26, *34 and *36), *TPMT* (*3A, *3B, *3C), and *G6PD* (Asahi and A-202A-376G). Python scripts for haplotype and diplotype assignment were used (see “Code availability” section, below). When available, we used parental variation data for phasing (i.e. *TPMT* *1/*3A or *3B/*3C haplotypes in 22 individuals heterozygous for rs1800460 and rs1142345; all found to be *TPMT* *1/*3A). For *G6PD*, located in the X-chromosome, the gender of the individual enabled to differentiate hemizygous males, homozygous females, and compound heterozygous females.

Resulting diplotypes were translated into their corresponding pharmacogenetic phenotypes following the functionality tables provided by CPIC clinical guidelines.

### Comparison of population-specific allele frequencies

Statistical analysis to compare the allele frequencies in our Spanish and Latin American subcohorts was performed using *χ*^2^ test. GnomAD variation data (number of alleles and minor allele frequencies) was extracted from MyVariant.info database^55^. Chi-square statistical analysis was performed to compare gnomAD Non-Finish European (NFE) and Latino/Admixed American (AMR) population data to our Spanish and Latin American individuals, respectively. The threshold for statistical significance was 0.001 due to multiple comparisons. Allele frequencies reported in CPIC guidelines were also used in the analysis.

### Discovery of novel variants

The variant discovery of novel and known variants (SNV and indel variants) in the 11 selected pharmacogenes was done using GATK v3.6. We selected all non-synonymous coding variants (e.g. missense, frameshift, stop gain, start lost) and those affecting canonical splice sites. A variant was considered as “novel” when it was not present neither in gnomAD (v2.1.1) nor in the SNP database (dbSNP v138-b37^56^). Novel frameshift, stop gain and start lost variants together with those altering canonical splicing sites were considered LOF. Variants with <20x depth of coverage or <30% Variant Allele Fraction were filtered out. However, novel variants with 10-20x coverage were manually curated.

### Reporting summary

Further information on research design is available in the Nature Research Reporting Summary linked to this article.

## Supplementary information

Supplementary Figures

Supplementary Data 1

Supplementary Data 2

Supplementary Data 3

Supplementary Data 4

Reporting Summary checklist

## Data Availability

Pharmacogenetics variation data to reproduce results presented in this work are deposited at the European Nucleotide Archive (ENA; https://www.ebi.ac.uk/ena/browser/home) and publicly available under accession number PRJEB48632. Novel variants are provided in Supplementary Data 3. Individual-related information (country and exome platform) are available in Supplementary Data 2.

## Code availability

Python scripts were created to perform the analyses described in the previous sections: coverage QC, genotyping data to CPIC alleles and phenotype conversion, collecting gnomAD population data for specific variants, all statistical analysis and filtering of the novel variants. Plots displayed in figures were also created using Python (v3.6). All scripts have been uploaded to a GitHub repository: https://github.com/jlanillos/clinAcc_PGx_WES.
