# Supplementary material for: Clinical pharmacogenetic analysis in 5,001 individuals with diagnostic Exome Sequencing data
Source: NPJ Genom Med. 2022 Feb 18;7:12. doi: 10.1038/s41525-022-00283-3 (PMC8857256; doi:10.1038/s41525-022-00283-3)
Supplement: Supplementary file 1 — Supplementary Figures [file 41525_2022_283_MOESM1_ESM.pdf]

SUPPLEMENTARY DATA CAPTIONS

Supplementary Data 1. Alleles and functional information from CPIC guidelines. All pharmacogenetic alleles described by CPIC guidelines in the 11 actionable genes described in this study: *CACNA1S, CYP2C9, CYP2B6, CYP4F2, DPYD, G6PD, NUDT15, RYR1, SLCO1B1, TPMT* and *UGT1A1*.

Supplementary Data 2. List of pharmacogenetic alleles detected in each individual included in the study.

Supplementary Data 3. Known and novel variants discovered in clinically actionable pharmacogenes studied.

Supplementary Data 4. Summary of available CPIC guidelines and exome repurposing in pharmacogenetics.

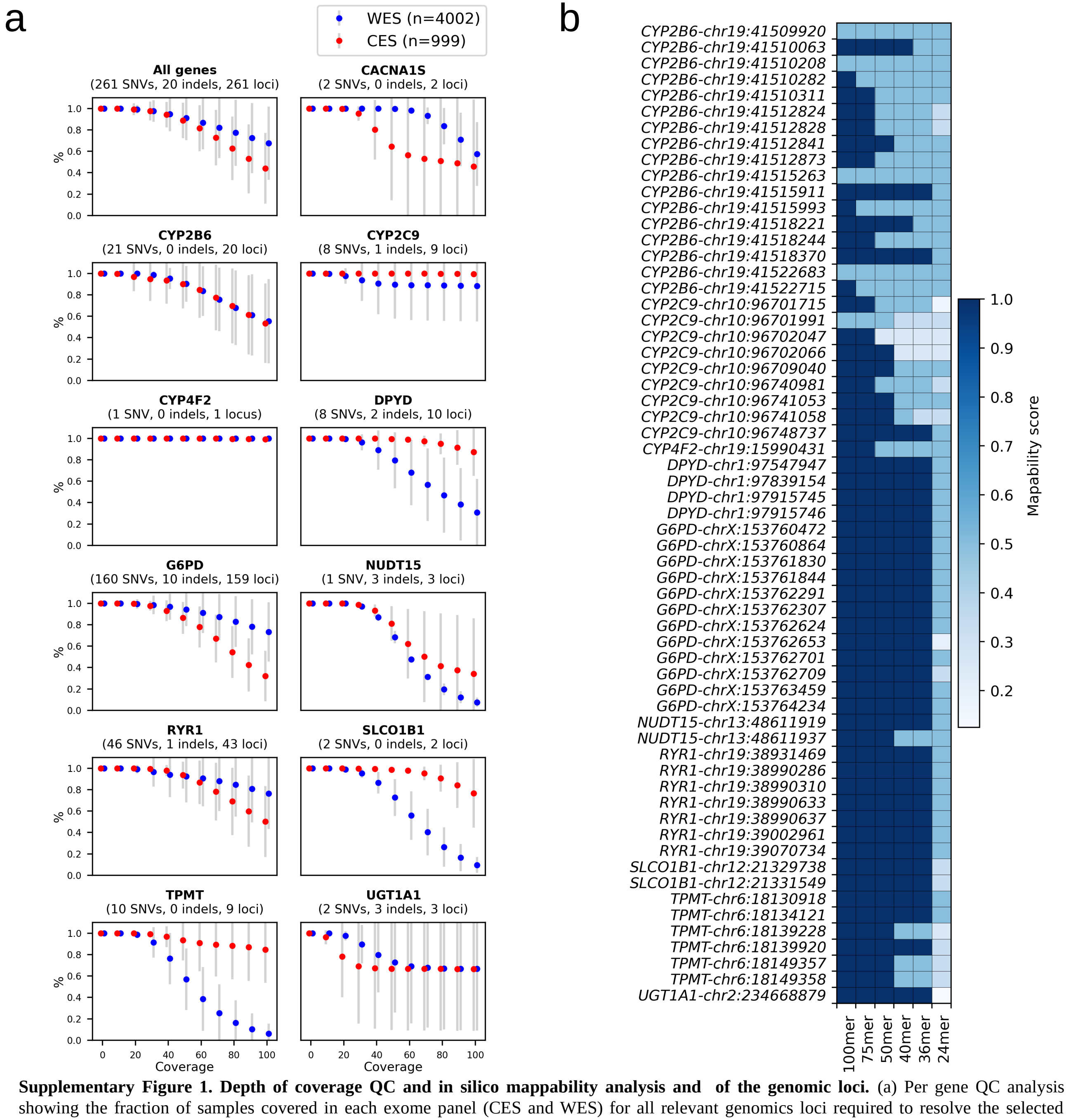

Supplementary Figure 1. Depth of coverage QC and in silico mappability analysis and of the genomic loci. (a) Per gene QC analysis showing the fraction of samples covered in each exome panel (CES and WES) for all relevant genomics loci required to resolve the selected actionable alleles. Error bars in grey show the standard deviation of each dot. (b) Mappability score for different k-mer window sizes (100-mer, 75-mer, 50-mer, 40-mer, 36-mer and 24-mer) were extracted from UCSC Mapability Tracks (see Methods). Only genomic loci with a mappability score <1 in any of the k-mer sizes are shown.

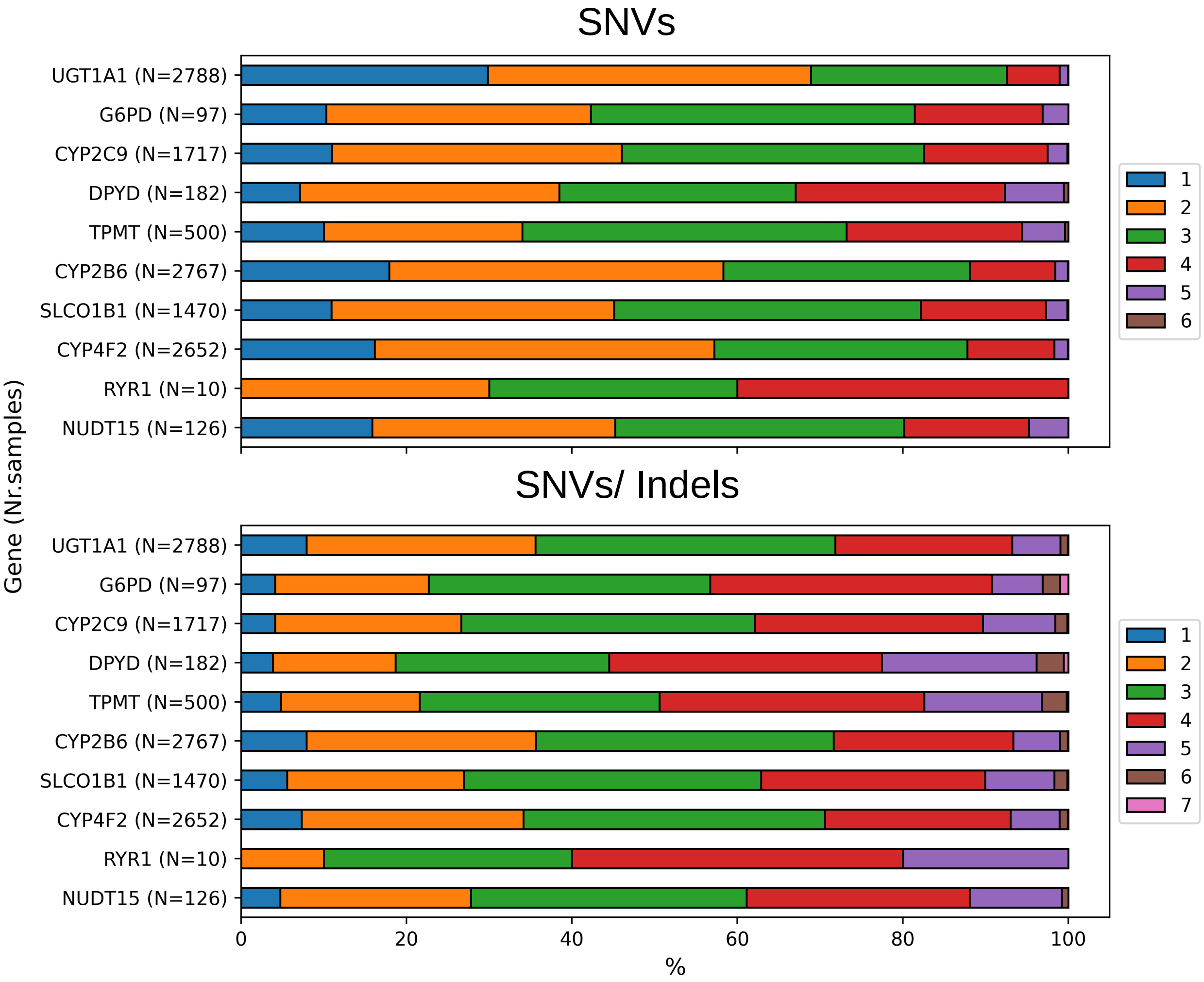

Supplementary Figure 2. Fraction of samples carrying one or more alleles per gene. Horizontal stacked bar plots representing the fraction of samples carrying a specific number of alleles. For example, for UGT1A1 carriers (upper or lower panel, first horizontal bar), orange color shows the fraction of UGT1A1 carriers which also carry another alleles.

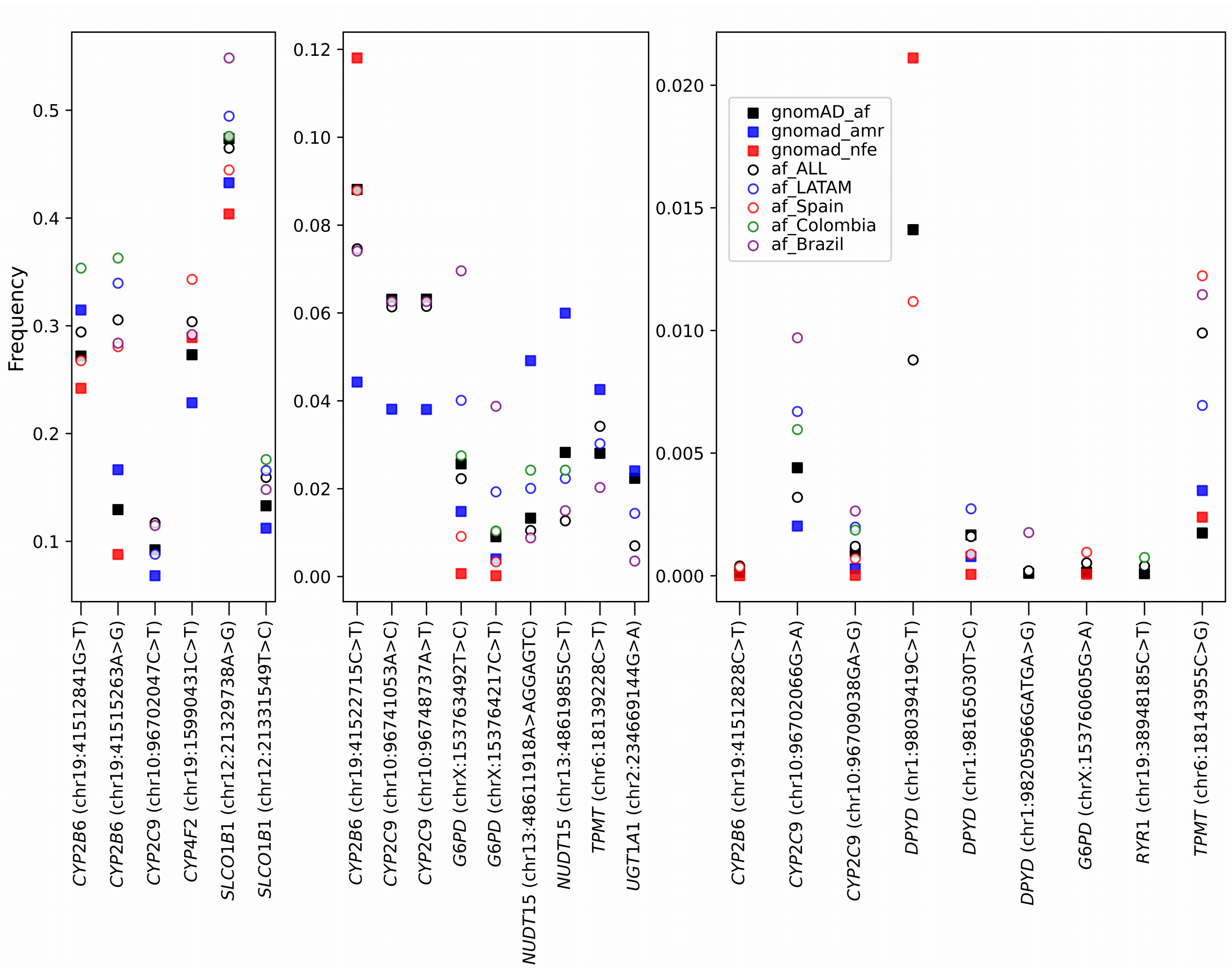

Supplementary Figure 3. Differences in variant frequencies between Spanish and Latin American individuals. Variant frequencies in our study (all individuals, Spain, Colombia and Brazil) and in their gnomAD reference populations (NFE and AMR). Only statistically significant comparisons by Chi-square test are shown. To enhance visualization, variants are splitted into three panels according to the variant frequencies. X-axis provides with gene name and variant.

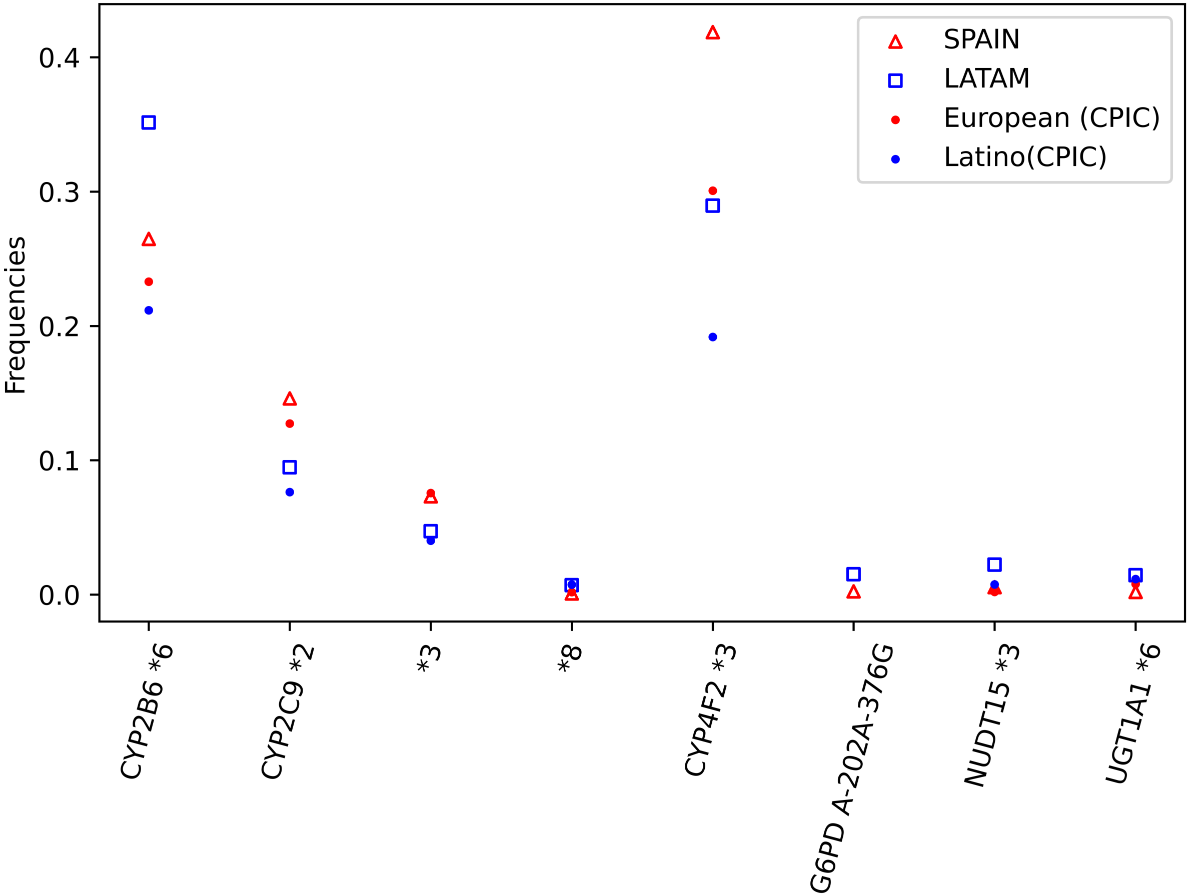

Supplementary Figure 4. Differences in allele frequencies between Spain and Latin American individuals. Only alleles with statistically significant differences by Chi-square test are shown. Spain (red triangles), Latin American countries (blue squares). Reported allele frequencies for “European” and “Latino” populations by CPIC (red and blue dots, respectively).

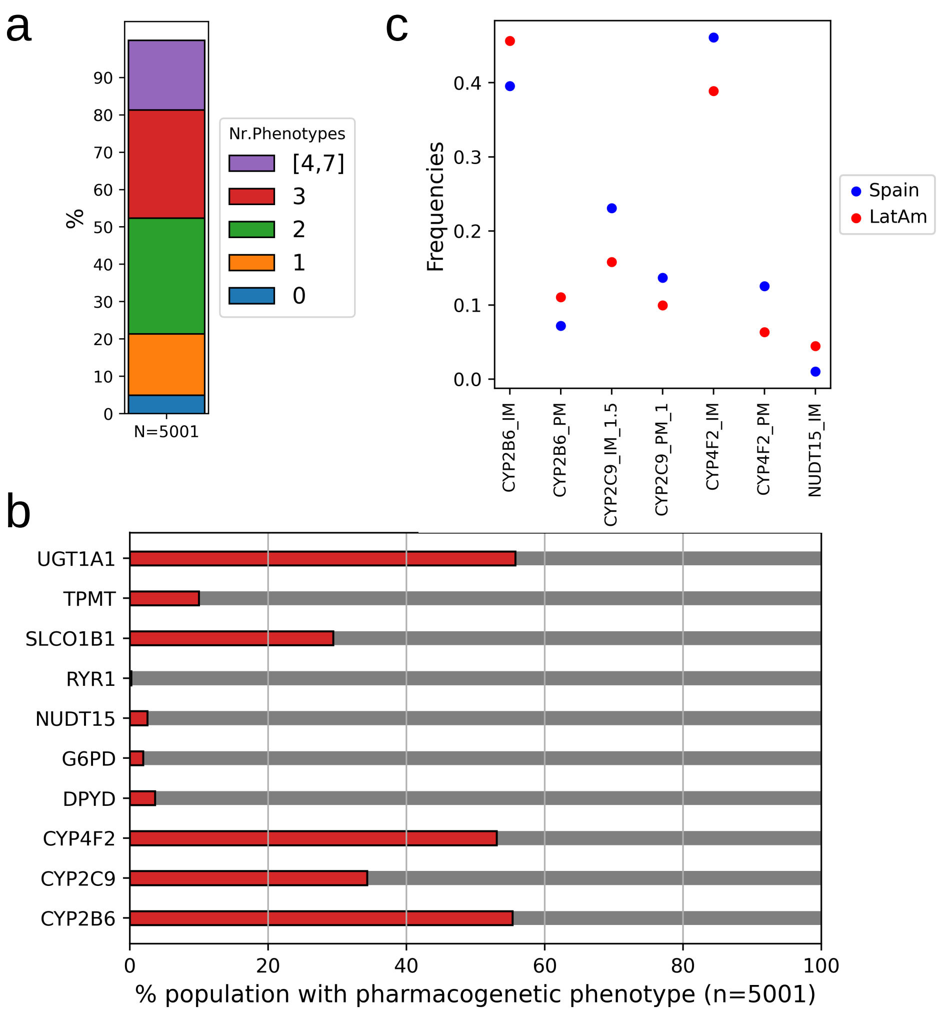

Supplementary Figure 5. Distribution of pharmacogenetic phenotypes. (a) Stacked vertical bar plot showing the percentage of individuals with zero, one, two, three or more than three pharmacogenetic phenotypes in the eleven genes chosen in the study. (b) Vertical bar plot showing the percentage of individuals with an actionable phenotype (*CACNA1S* is not shown, since no variants in this gene were found). (c) Phenotype frequencies comparison between individuals sequenced in Spain (red dot) versus individuals sequenced in Latin American countries (blue dot). Only phenotypes with statistically significant differences by Chi-square test are shown.
